# Intravenous Misplacement of the Nephrostomy Catheter Into the Renal Vein Following Percutaneous Nephrolithotomy (PCNL): A Case Report and Literature Review

**DOI:** 10.1155/crin/1229960

**Published:** 2025-05-19

**Authors:** Shuah Ullah, Hammad Ur Rehman Shamsi, Ayesha Kamran, Abdullah Ayub, Dania Masood

**Affiliations:** ^1^Sindh Institute of Urology and Transplantation, Karachi, Pakistan; ^2^Dow University of Health Sciences, Karachi, Pakistan

**Keywords:** nephrostomy tube misplacement, percutaneous nephrolithotomy (PCNL), post-PCNL complications, renal vein perforation

## Abstract

**Background:** After percutaneous nephrolithotomy (PCNL), intravenous misplacement of a nephrostomy tube is a very rare clinical occurrence. This report summarizes the characteristics and management of intravenous misplacement of a nephrostomy tube.

**Case Presentation:** We present a rare case of intravenous nephrostomy catheter misplacement after PCNL in a 63 years old male. The tip of the tube was located in the left renal vein. The patient was managed conservatively and treated safely.

**Conclusion:** Intravenous nephrostomy tube misplacement is a rare PCNL complication. Good Imaging can rule out through and through renal vein perforation and thus patients can be safely managed using conservative approach.

## 1. Introduction

Percutaneous nephrolithotomy (PCNL) was first introduced by Fernstrom and Johansson in 1976 [1]. It has become as a method to temporarily alleviate the obstruction, infection, and dilation of the kidney and is also the treatment of choice for renal calculi [2]. There are two mandatory steps of PCNL, i.e., puncturing and dilation. Rarely, during dilatation, dilators can cause direct injury to the main renal vein or its tributaries [3]. A nephrostomy tube is routinely placed after PCNL in the renal pelvis in order to control bleeding and drain the collecting system. Although it is a well-established procedure but complication rates of up to 7% have been reported [4]. Intraoperative complications include bleeding, damage to visceral organs, injury to the renal collecting system, pulmonary complications, extra-renal migration of stone fragments, thromboembolic disorders, and incorrect placement of the nephrostomy tube. On the other hand, postoperative complications include infection, persistent urinary fistula, sepsis, bleeding, infundibular stenosis, and, in severe cases, nephrectomy and patient mortality [4]. Therefore, the mechanism and cause of injury should be investigated properly. We report a case of misplacement of nephrostomy tube, who underwent PCNL.

## 2. Case Presentation

**Figure 1:**
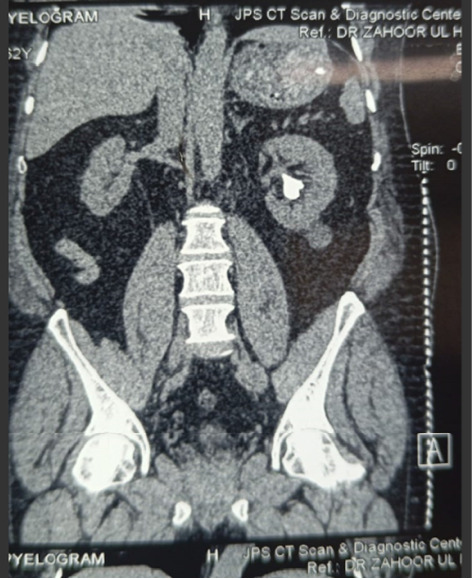
Pre-op CT.

**Figure 2:**
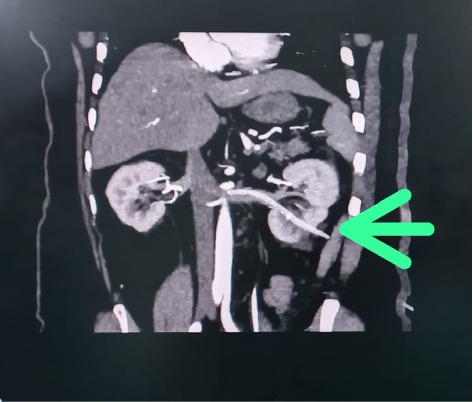
CT indicating nephrostomy tube in left renal vein.

**Figure 3:**
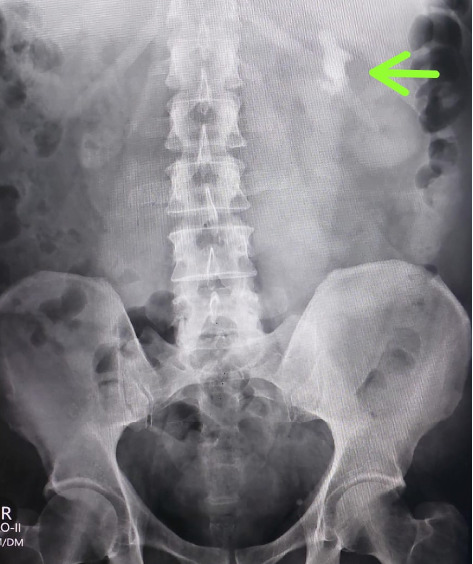
Pre-op x-ray showing left renal stone.

**Figure 4:**
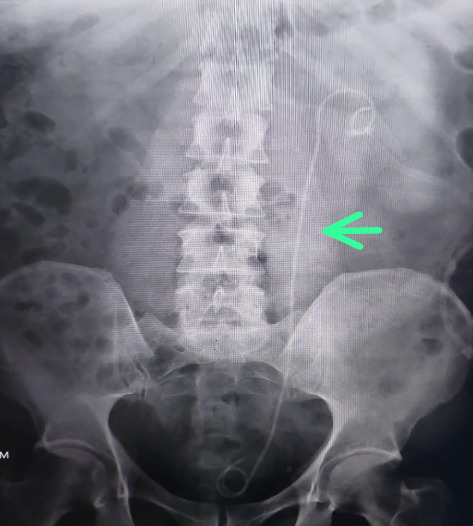
Post-op x-ray showing left stone free with DJ stunt in situ.

A 63-year-old male with no known comorbidities presented with a six-month history of left flank pain and lower urinary tract symptoms. Radiological investigations revealed renal calculi in the left kidney as shown in Figures 1, 2 and 3 indicating a need for PCNL. The procedure was performed, and a DJ stent was placed for postoperative drainage as shown in Figure 4. Additionally, a nephrostomy tube was inserted for drainage purposes.

**Figure 5:**
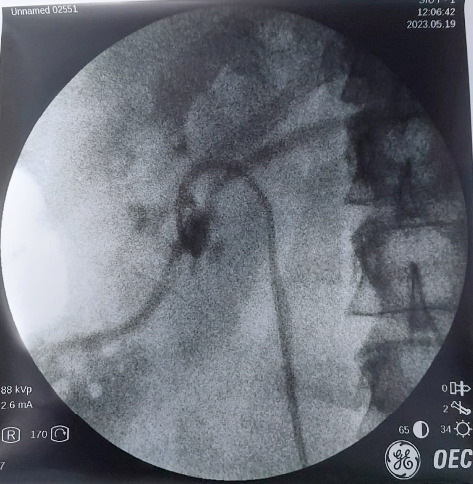
Antegrade left pyelogram, contrast can be seen going into renal vein.

The patient experienced intense bleeding after 5 h following the nephrostomy tube insertion, which subsequently resulted in hypotension later on. He was transferred to the High Dependency Unit (HDU), for resuscitation. Four units of blood along with fresh frozen plasma (FFP) were transfused, stabilizing the patient's hemodynamics. On further radiological imaging, specifically a CT angiogram, showed that the nephrostomy tube had accidently traversed the lower pole calyces of the left kidney, reaching the left renal vein, with its tip positioned only 2 mm away from the inferior vena cava (IVC) ostium (Figure 5). Patient was kept stabilized through fluid resuscitation and by keeping the PCN tube clamped.

The patient was taken back to the operating theatre to address the cause of bleeding on the second postoperative day. The nephrostomy tube was withdrawn from the renal vein under fluoroscopic guidance, and the plan was to keep it in the kidney pelvicalyceal system. However, due to a nonhydronephrotic system, it came out completely from the kidney, so it was kept as a drain for 24 h. The patient remained vitally stable afterward, and minimal hematuria was observed in the Foley's catheter, which resolved with hydration alone. Serial ultrasounds were performed at intervals of 6 h, all of which were unremarkable. Subsequent monitoring showed stable hemodynamics as shown in Tables 1 and 2, and no further bleeding episodes occurred.

## 3. Discussion

PCNL is now considered a standard procedure for patients with renal stones. The common complications after PCNL include sepsis, iatrogenic injury to adjacent structures (such as the liver, spleen, and bowel), failed renal access, perforation of the renal pelvis, pleural effusion, and pneumothorax [5]. The most significant complication associated with this procedure is hemorrhage. Severe bleeding can occur due to needle puncture, tract dilation, or nephrostomy tube placement [3]. The close association of the renal veins with the renal pelvis and major posterior calyces makes them susceptible to damage during the steps of PCNL [6].

In this case, PCNL was performed for renal calculi, and the nephrostomy tube was inserted for drainage of the collecting system. A DJ stent was placed to maintain the patency of the ureter after the procedure. Five hours after the placement of the nephrostomy tube, the tube was unclamped to observe urine output and the passage of residual stones. Upon unclamping, gross hematuria of approximately 500–1000 mL was observed. At that time, the patient was stable, so the PCN tube was kept clamped, and the patient was closely monitored alongside fluid replacement. Upon unclamping the PCN tube for the second time, similar gross hematuria with a blood loss of 500–1000 mL was observed. The patient subsequently became hemodynamically unstable and was shifted to the HDU, where 4 units of packed cell volume (PCV) and FFP were administered to maintain hemodynamics. Investigations were performed to identify the possible complications. An ultrasound was performed, but no significant findings were observed. On CT angiography, the nephrostomy tube was found to be in the renal vein, with its tip only 2 mm away from the IVC (as shown in Figure 5).

There are two possible ways to manage a misplaced nephrostomy tube: conservative and surgical. In our case, the patient was successfully managed using conservative treatment. In cases involving misplacement of a nephrostomy tube, depending on the postoperative time elapsed, nephrostomy tube transposition is advised under fluoroscopic guidance, and the surgical team should be prepared for emergency surgery if required [7]. Using fluoroscopic guidance with contrast, the nephrostomy tube was completely withdrawn, as the patient was hemodynamically and vitally stable. Repeated blood tests (Tables 1 and 2) and ultrasounds were performed. The patient developed slight hematuria, which was detected through the Foley's catheter, but it was managed by hydrating the patient under the cover of a diuretic. The patient was kept under observation for 48 h to monitor for any possible recurrent hematuria, but none occurred. The patient was discharged successfully. The data from these and other similar publications are summarized in Table 3.

## Figures and Tables

**Table 1:** CBC reports.

Tests	Normal ranges	Unit	Immediately after surgery	8 h postsurgery	16 h postsurgery
HGB	13–16.5	g/dL	9.4	7.6	8.6
HCT	40–52	%	28.7	23.4	35.8
RBC	4.5–6.5	10^12^L	3.14	2.57	2.97
PLATELETS	150–400	10^9^/L	187	183	147
WBC	4.0–11.0	10^9^/L	11.56	12.88	15.99

**Table 2:** Urea-creatinine-electrolyte (UCE) report.

Tests	Results units (on the day of procedure) (mg/dL)	Results units (4 days after the procedure) (mg/dL)	Reference range (mg/dL)
Urea serum	25	23	15–39
Creatinine Serum	0.9	0.83	0.5–1.5

**Table 3:** Literature review.

Author	Age/sex	Operation type	Catheter type	Location	Catheter withdrawal	Subsequent treatment
Dias Filho et al. [8]	63/F	PCNL	Foley catheter	Left renal vein	1-step under fluoroscopy	Second PCNL
Wah et al. [9]	54/M	PCNL	Nephrostomy tube	Renal vein	2-step under fluoroscopy	Exploratory
Mazzucchi et al. [7]	52/M	PCNL	Nephrostomy tube	Renal vein	1-step under fluoroscopy	No
Mazzucchi et al. [7]	35/M	PCNL	Nephrostomy tube	Renal vein, IVC	2-step under fluoroscopy	No
Li et al. [10]	32/M	PCNL	Nephrostomy tube	Renal vein, IVC	2-step under fluoroscopy	No
Wang et al. [11]	66/M	PCNL	Nephrostomy tube	Renal vein	1-step under fluoroscopy	No
Kotb et al. [6]	50/M	PCNL	Foley catheter	Renal vein, IVC	1-step open pyelotomy	Open pyelotomy
Chen et al. [12]	42/M	PCNL	Nephrostomy tube	Renal vein, IVC	2-step under CT guide	Second PCNL
Chen et al. [12]	38/F	PCNL	Nephrostomy tube	Renal vein, IVC	2-step under fluoroscopy	Simultaneous PCNL
Chen et al. [12]	48/M	PCNL	Nephrostomy tube	Renal vein	1-step under ultrasound	Second PCNL

## Data Availability

The data supporting the findings of this study are available within the article. Any additional data or materials can be made available upon reasonable request from the corresponding author.

## Nomenclature

CT: Computed tomography
IVC: Inferior vena cava
PCNL: Percutaneous nephrolithotomy

## References

[1] Fernström I., Johansson B. (1976). Percutaneous Pyelolithotomy. A New Extraction Technique. *Scandinavian Journal of Urology and Nephrology*.

[2] Ganpule A. P., Vijayakumar M., Malpani A., Desai M. R. (2016). Percutaneous Nephrolithotomy (PCNL) a Critical Review. *International Journal of Surgery*.

[3] Mahmood S. N., Toffeq H. M. (2016). Renal Vein Injury During Percutaneous Nephrolithotomy Procedure. *Journal of Endourology Case Reports*.

[4] Lingeman J. E. (2007). Campbell-walsh Urology. *Urinary lithiasis and endourology*.

[5] Srivastava A., Singh K. J., Suri A. (2005). Vascular Complications after Percutaneous Nephrolithotomy: Are There Any Predictive Factors?. *Urology*.

[6] Kotb A. F., Elabbady A., Mohamed K. R., Atta M. A. (2013). Percutaneous Silicon Catheter Insertion into the IVC, Following Percutaneous Nephrostomy (PCN) Exchange. *Canadian Urological Association Journal*.

[7] Mazzucchi E., Mitre A., Brito A., Arap M., Murta C., Srougi M. (2009). Intravenous Misplacement of the Nephrostomy Catheter Following Percutaneous Nephrostolithotomy: Two Case Reports. *Clinics*.

[8] Dias-Filho A. C., Coaracy G. A., Borges W. (2005). Right Atrial Migration of Nephrostomy Catheter. *International Brazil Journal Urology*.

[9] Wah T. M., Kellett M. J., Choong S., Choong S. K. (2005). Management of Renal-Vein Perforation During a Challenging Percutaneous Nephrolithotomy. *Journal of Endourology*.

[10] Li D., Xiao L., Tang Z. (2013). Management of Intravenous Migration of Urologic Catheter. *Urology*.

[11] Wang C., Chen S., Tang F., Shen B. (2013). Metachronous Renal Vein and Artery Injure After Percutaneous Nephrostolithotomy. *BMC Urology*.

[12] Chen X. F., Chen S. Q., Xu L. Y., Gong Y., Chen Z. F., Zheng S. B. (2014). Intravenous Misplacement of Nephrostomy Tube Following Percutaneous Nephrolithotomy: Three New Cases and Review of Seven Cases in the Literature. *International Brazil Journal Urology*.

